# Using a social marketing framework to evaluate recruitment of a prospective study of genetic counseling and testing for the deaf community

**DOI:** 10.1186/1471-2288-13-145

**Published:** 2013-11-25

**Authors:** Yoko Kobayashi, Patrick Boudreault, Karin Hill, Janet S Sinsheimer, Christina GS Palmer

**Affiliations:** 1 Department of Deaf Studies, California State University, Northridge, California, USA; 2 Department of Biostatistics, University of California, Los Angeles, California, USA; 3 Department of Biomathematics, University of California, Los Angeles, California, USA; 4 Department of Human Genetics, University of California, Los Angeles, California, USA; 5 Institute for Society and Genetics, University of California, Los Angeles, California, USA; 6 Department of Psychiatry & Biobehavioral Sciences, University of California, Los Angeles Semel Institute 760 Westwood Plaza Room 47-422, Los Angeles, California 90024, USA; 7 Current affiliation, Department of Health Service Research, University of Tsukuba, Tsukuba, Ibaraki, Japan; 8 Current affiliation, Gallaudet University, Washington DC, USA

**Keywords:** Social marketing, Genomic medicine, Deaf, Deaf, Hard-of-hearing, American Sign Language, Genetic testing, Health disparity, Health service research, Minority groups, Communities, Study design

## Abstract

**Background:**

Recruiting deaf and hard-of-hearing participants, particularly sign language-users, for genetics health service research is challenging due to communication barriers, mistrust toward genetics, and researchers’ unfamiliarity with deaf people. Feelings of social exclusion and lack of social cohesion between researchers and the Deaf community are factors to consider. Social marketing is effective for recruiting hard-to-reach populations because it fosters social inclusion and cohesion by focusing on the targeted audience’s needs. For the deaf population this includes recognizing their cultural and linguistic diversity, their geography, and their systems for information exchange. Here we use concepts and language from social marketing to evaluate our effectiveness to engage a U.S. deaf population in a prospective, longitudinal genetic counseling and testing study.

**Methods:**

The study design was interpreted in terms of a social marketing mix of Product, Price, Place, and Promotion. Price addressed linguistic diversity by including a variety of communication technologies and certified interpreters to facilitate communication; Place addressed geography by including community-based participation locations; Promotion addressed information exchange by using multiple recruitment strategies. Regression analyses examined the study design’s effectiveness in recruiting a culturally and linguistically diverse sample.

**Results:**

271 individuals were enrolled, with 66.1% American Sign Language (ASL)-users, 19.9% ASL + English-users, 12.6% English-users. Language was significantly associated with communication technology, participation location, and recruitment. Videophone and interpreters were more likely to be used for communication between ASL-users and researchers while voice telephone and no interpreters were preferred by English-users (Price). ASL-users were more likely to participate in community-based locations while English-users preferred medically-based locations (Place). English-users were more likely to be recruited through mass media (Promotion) while ASL-users were more likely to be recruited through community events and to respond to messaging that emphasized inclusion of a Deaf perspective.

**Conclusions:**

This study design effectively engaged the deaf population, particularly sign language-users. Results suggest that the deaf population’s cultural and linguistic diversity, geography, and forms of information exchange must be taken into account in study designs for successful recruitment. A social marketing approach that incorporates critical social determinants of health provides a novel and important framework for genetics health service research targeting specific, and hard-to-reach, underserved groups.

## Background

This article evaluates the Deaf Genetics Project’s (DGP) degree of success at motivating a hard-to-reach, underserved population – ranging from culturally Deaf to hard-of-hearing individuals – to participate in a genetic counseling and test study through a social marketing framework that addresses critical social determinants of health: social inclusion and social cohesion. Although recruiting individuals in underserved populations into health service research studies is problematic [1-3], recruiting deaf^a^ participants for health service research is even more challenging due to this population’s cultural and linguistic diversity, communication barriers, literacy level, mistrust, and researchers’ unfamiliarity with deaf people [4-7]. Researchers in previous studies have addressed these issues in various ways by building relationships with deaf organizations [8-11], receiving cultural sensitivity or awareness training [8], ensuring that research procedures, e.g., questionnaires or focus groups, are accessible in the participants’ language [8-11], and more recently, by involving deaf individuals in the research process from study design to data interpretation [12]. However, there are little empirical data on effective strategies to promote deaf individuals’ participation in health service research [13,14], and no data directly related to participation in genetics health service research. Therefore, the deaf population is a group least addressed in genetics health promotion and health care delivery efforts. However, we anticipate more genetic services devoted to common adult conditions, such as cancer, heart disease and diabetes; and with personalized genomic medicine everyone may benefit from genetic services in their lifetime. Therefore, research on effective methods for motivating deaf individuals to participate in genetics health service research is essential for reducing health disparities.

For many within the U.S. culturally Deaf community, American Sign Language (ASL) is the primary language and English (either spoken or written) is the second language. However, language preference, mode of communication, and identities/cultural affiliation (culturally Deaf, culturally hearing, bicultural) vary within the deaf population [15] due to factors such as experiences interacting with deaf and hearing individuals, amount of residual hearing, upbringing, and school setting [16], such that spoken English is some individuals’ primary language. To a large degree, ASL-users constitute a linguistic and cultural minority group who view their deafness as human variation [17,18]. As with other linguistic and cultural minorities, institutional, socio-cultural, and educational factors affect sign language-users’ knowledge, acceptance, and use of health-related information and services [4]. Many health researchers face difficulties working with sign language-users due to these cultural and linguistic differences [7,19]. Moreover, when developing effective strategies to promote genetic counseling and testing, or genomic medicine more generally, researchers must consider often overlooked barriers experienced by sign language-users, such as inadequate science education and few opportunities for incidental learning of genetics terms [4,20,21] that create challenges understanding genetics concepts. Importantly, researchers must also consider the Deaf community’s general distrust towards genetics due to perceptions of cultural insensitivity and the history of eugenics [22].

Social marketing is effective for recruiting underserved or hard-to-reach populations into research [3,23]. In this context, commercial marketing tools facilitate voluntary behavior change for the benefit of individuals, groups, or society rather than commercial gain [24,25]. Social marketing focuses on the targeted audience’s needs, which for the deaf population includes recognizing their cultural and linguistic diversity, the geography of Deaf communities, and that sign language-users are a collective group [18] who use an alternate networking system for information exchange [26]. Importantly, social marketing techniques increase both social inclusion – the ability for a community, such as the Deaf community, to be included in social systems and relationships [27] -- and social cohesion – the degree to which values, a sense of connectedness, trust and familiarity are shared among a group [28]. Programs that increase these factors improve a community’s well-being [27-29].

Here we describe our study design and interpret our effectiveness at motivating deaf individuals, particularly sign language-users, to participate in this research through a social marketing framework that emphasizes social inclusion and social cohesion. To our knowledge, this is the only study to examine outcomes of genetic counseling and testing in the deaf population; hence successful recruitment and retention were essential for generating data to fill the gaps in knowledge. More generally, by interpreting our study’s recruitment through a social marketing perspective, our results suggest that a social marketing approach can provide a novel and important framework for genetics health service research hoping to target specific, and hard-to-reach, underserved groups such as the deaf population in general and especially the Deaf community who use sign language.

## Methods

### Overview of the deaf genetics project

The Deaf Genetics Project was a prospective, longitudinal study to examine the impact of genetic counseling and genetic testing on deaf adults and the Deaf community. The study focused on testing two deaf genes, *GJB2* and *GJB6* (also called by their protein products, Connexin 26 and Connexin 30, respectively), which for some deaf individuals provides a genetic explanation for why they are deaf. The Deaf Genetics Project was designed by a multi-institutional, multi-disciplinary team of Deaf, hard-of-hearing and hearing investigators. Research staff included audiologists and board-certified genetic counselors. Among the four audiologists, one was a certified sign language interpreter, one was familiar with ASL, and two were not familiar with ASL. None of the four genetic counselors were able to communicate directly with participants using ASL. Thus, three certified ASL/English interpreters also were members of the project staff, and an interpreter was available for all participants during the course of the research protocol unless the participant opted to use spoken English without an interpreter. The goals of the DGP were to enroll 250 deaf individuals in ~2 year period, to determine for each participant if they had *GJB2* or *GJB6* genetic deafness, and to understand the impact of this genetic information on participants. Individuals who were at least 18 years old with an unexplained sensorineural deafness since an early age (defined as birth to age 6 years) were eligible to participate. Participants were recruited over a period of 25 months from the Los Angeles, Bay Area, and Riverside areas of California.

**Figure 1:**
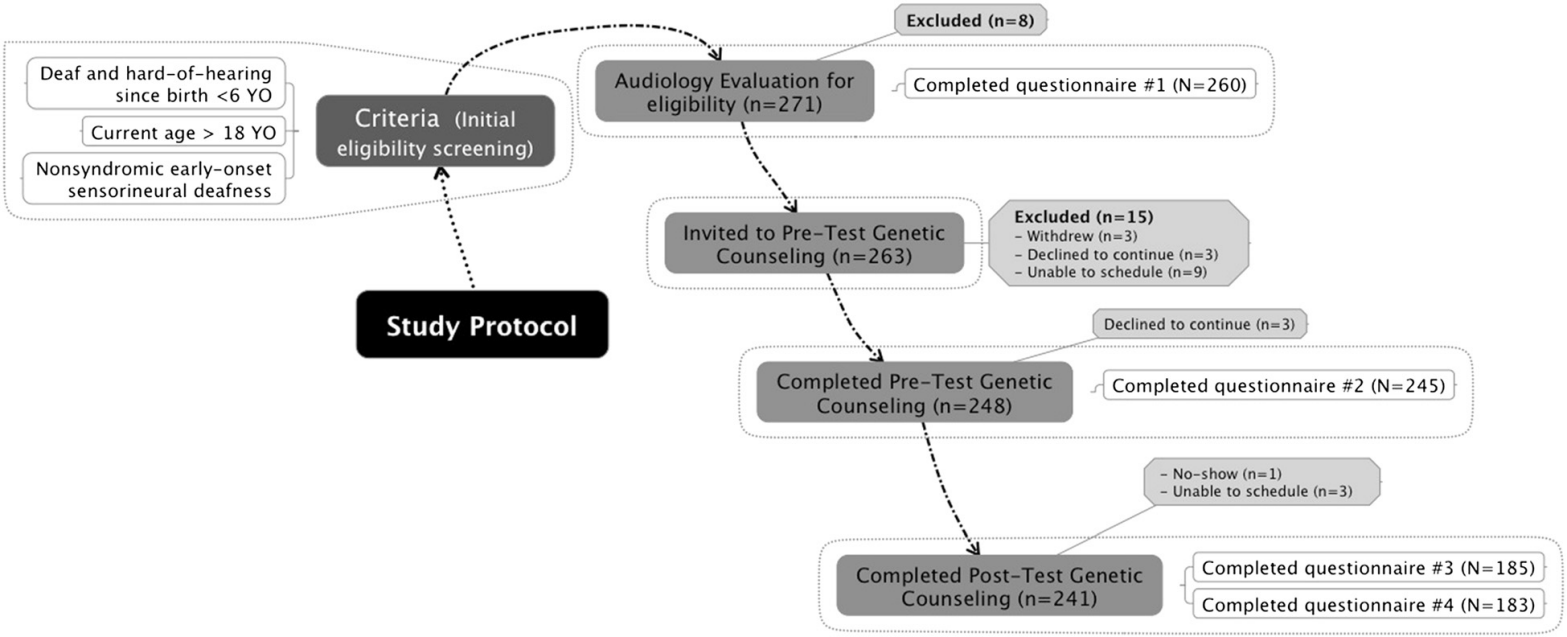
**Deaf genetics project study protocol.** Note: YO = years old.

The study protocol involved three steps: 1) audiology session to confirm sensorineural deafness, 2) pre-test genetic counseling to obtain informed consent for genetic testing, and 3) post-test genetic counseling to disclose genetic test results. For all three stages, individuals selected one of four locations for their participation: University of California Los Angeles (UCLA), California State University Northridge (CSUN), California School for the Deaf-Fremont (CSDF), or California School for the Deaf-Riverside (CSDR). Participants determined eligible to participate in steps 2 and 3 were asked to complete a series of four questionnaires: following audiology, following pre-test genetic counseling, and 1-month and 6-months following disclosure of genetic test results. The questionnaires assessed demographic factors, reasons for genetic testing, attitudes toward genetic testing, knowledge and understanding of genetics and genetic testing, cultural affiliation and deaf identity, and a variety of psychological and behavior measures. Participants were informed that DNA samples would only be used for testing the *GJB2* and *GJB6* genes. All DNA samples were discarded at the end of the study. This research study was conducted in compliance with the Helsinki Declaration and approved by the institutional review board at the University of California Los Angeles (#10-001193) and by the Committee for Protection of Human Subjects at the California State University-Northridge. Details on the participants and study protocol are depicted in Figure 1 and also have been published elsewhere [30-32].

### Overview of the social marketing framework

**Figure 2:**
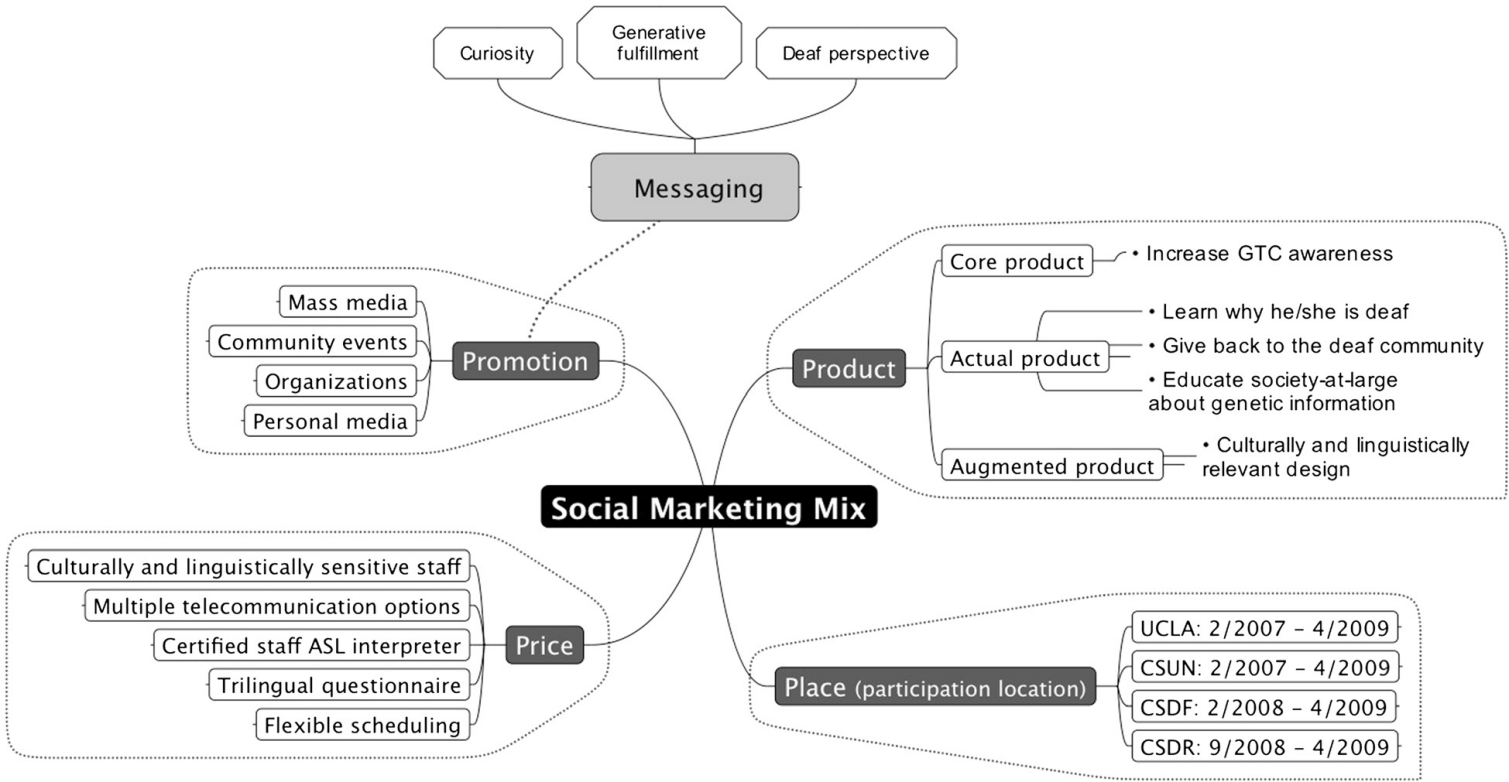
**Social marketing framework used by deaf genetics project.** Note: GTC = genetic testing and counseling; UCLA = University of California Los Angeles; CSUN = California State University Northridge; CSDF = California School for the Deaf-Fremont; CSDR = California School for the Deaf-Riverside.

In this section we describe how the DGP study design can be viewed through a social marketing mix of product, price, place, and promotion [24,25,33-38] (depicted in Figure 2).

#### Product

Following Kotler & Lee [39], we divided product into 3 parts. Core product, which is what people gain when they perform the behavior or the desired benefit from the *public health perspective*, was to increase genetic counseling and testing awareness in the deaf population. Actual product is the desired behavior or benefit from the potential *participant’s perspective.* Because conveying the significance of genetic information is key to motivate participation and decision making [40], our actual product was multi-pronged: learn why he/she is deaf, give back to the Deaf community, and educate society-at-large about what genetic information means to them. Augmented product is the tangible objects and services used to facilitate change. By applying a multi-layered cultural accessibility approach to the study design, our augmented product is the improvement in health service for deaf individuals and the Deaf community that occurs when providers are culturally competent and barriers to access are reduced [41]. Our multi-layered cultural accessibility approach was facilitated by our multidisciplinary team composed of Deaf, hard-of-hearing, and hearing researchers who worked to ensure the study design’s appropriateness and to increase the Deaf community’s trust [6].

#### Price

Price is the cost that the target market associates with adopting the desired behavior [39]. An important consideration for deaf individuals is the psychological cost associated with communication -- within medical settings, with healthcare providers, and with researchers [41]. Importantly, this cost increases social exclusion and decreases social cohesion between participants and researchers. However, our use of multiple communication approaches to embrace a variety of communication modes fostered social inclusion and cohesion and minimized this cost. Videophone, text telephone (TTY), and instant messaging, which are not prevalent in health care settings, as well as voice telephone and email, were available in the DGP research setting for enabling distance contact between project personnel and participants. Research staff included 3 certified ASL/English sign language interpreters who were available for in-person sessions. The interpreters were trained to convey complex genetics terminology and concepts with lay and visual terms in ASL to ensure high quality and consistent interpreting across participants. They were bilingual and bicultural, and their presence during sessions helped ensure clear and appropriate communication between the genetic counselor and the participant [42]. The genetic counselors were trained in cultural sensitivity which enabled Deaf participants to raise questions without having to explain or educate the professionals about their condition and values. Questionnaires were translated into ASL and available in ASL (video streaming), English text, and bilingual format of ASL video + English text.

#### Place

Place is where and when the target behavior is performed and is an underused social marketing component [43]. However, place can be considered an essential component of our enhancement of social inclusion because in our study design we recognized that sign language-users prefer to reside near educational opportunities for deaf individuals, creating Deaf communities where residential schools or mainstreaming programs are located. Thus, we developed three community-based participation locations (CSDF, CSDR, CSUN), and one medically-based location (UCLA).

#### Promotion

The purpose of “Promotion” is to inspire action in target audiences [39]. In a social marketing framework, the DGP’s use of branding, messaging, and recruitment channels can be considered promotion techniques to recruit participants in the following ways.

##### Branding

**Figure 3:**
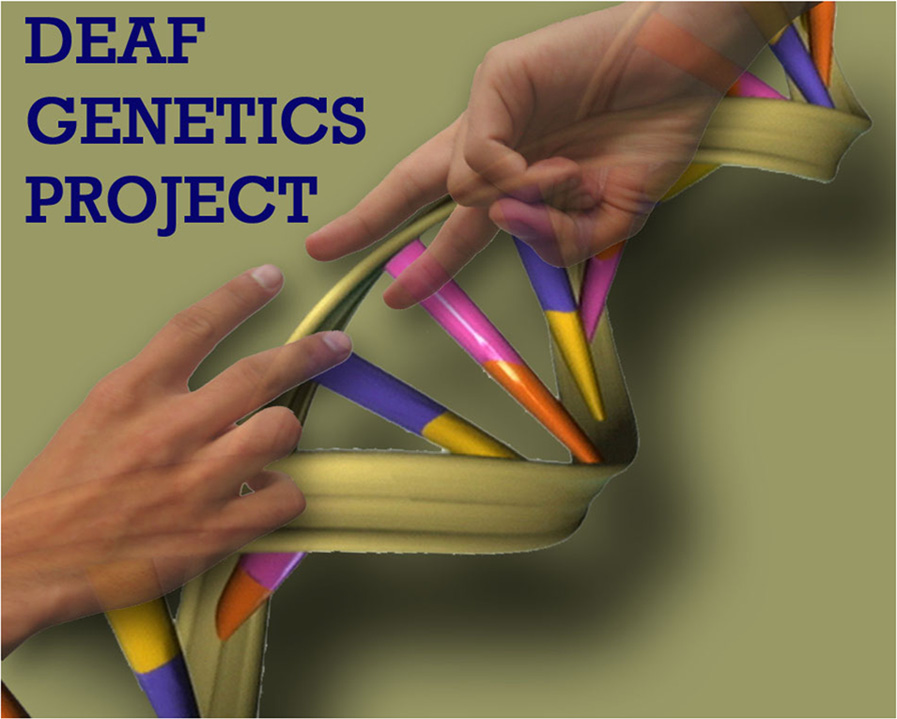
Deaf genetics project logo.

The name “Deaf Genetics Project” with the image of a DNA helix overlayed with two hands signing DNA (Figure 3) was developed to uniquely identify our study and resonate with many deaf individuals regardless of their attitude toward genetics and genomics health service research. The brand identity provided a look and feel that helped to unify and integrate all study activities. To maximize impact and effectiveness, the “Deaf Genetics Project” brand and logo were used on all study materials, including website, brochures, postcards, and flyers.

##### Messaging

Messaging strategies are an essential component of social marketing and are a widely used health communication campaign [44]. Our development of two primary messages to inform the deaf population about the study themes, promote study activities, and to inspire participation can be considered messaging. One message focused on an individual’s *curiosity* to learn why they are deaf. Another message focused on *generative fulfillment*[23], i.e., sharing their experience could educate society about the impact of genetic testing on the Deaf community. Secondary messages endorsed social cohesion and included that the team was composed of Deaf, hard-of-hearing, and hearing researchers, and that genetic testing was not intended to cure deafness or to affect an individual’s hearing [30]. This messaging was meant to reassure Deaf individuals that our goal was to benefit the Deaf community, not threaten it --- a goal recently reiterated in McKee et al. [7]. Importantly, positive framing was used [45]. Recruitment materials and DGP staff intentionally used terms “deaf” or “hard-of-hearing” instead of “hearing loss” or “hearing impaired;” “chance” instead of “risk;” “variant” instead of “mutation;” and avoided “normal” or “abnormal.”

##### Recruitment channels

Four recruitment channels were used: mass media, community events, organizations, and personal media. All recruitment strategies encouraged interested individuals to directly contact DGP personnel, or to allow DGP staff to contact them (with their permission) to initiate the study protocol process.

For recruitment through mass media, the DGP developed a website (http://www.deafgeneticsproject.org), multicolored brochure, multicolored visual postcard, and black and white flyer. Because deaf individuals generally prefer a visual-oriented approach, the DGP primarily used a visual discourse model, which fostered social inclusion. Recruitment items included the DGP logo and messaging to disseminate information about the project to the target population and to people involved with the deaf population via internet, mass mailings, listservs, and displays. The website provided information with ASL videos and English text to increase accessibility. Information on the website included DGP’s goals, eligibility criteria, procedures, frequently asked questions, staff, directions, and outreach efforts. English terms were simple and we used written lay terms to ensure literacy accessibility to the deaf population, for whom English is a second language [46]. A link to the website’s URL was included in messages through listservs to reach many people at once [38]. The multicolored brochure included the DGP logo, the purpose of DGP, criteria, estimated outcome, possible benefits and risks of participating, frequently asked questions, contact information, and DGP’s website address. The multicolored visual postcard used more pictures and simpler text messaging than the brochure, and included only the DGP logo, purpose, and website address. The black and white flyer included the study purpose, location, criteria, research procedures, and contact information. A variety of mass media approaches were used to complement the strengths and weaknesses of each approach. As one example, brochures and postcards can convey in-depth information, especially about complex issues, often at low cost, and often promote follow-up by interested individuals to request more information [38]. As another example, flyers are useful for generating awareness using limited amount of information, and can be placed in high-visibility places. Except for the study website, mass media approaches were used only in the UCLA, CSUN, and CSDF geographic locations.

A culturally relevant recruitment channel is *community events* regularly attended by Deaf individuals, e.g., Deaf Festivals, DeafNation Expos (http://www.deafnation.com), or social events sponsored by deaf organizations such as homecoming festivals at deaf residential schools. Community events allowed the DGP to staff an exhibit booth and disseminate information in multiple direct and interactive ways in an informal, brief period using a structured message. As part of the messaging kit, the booth included a banner with DGP logo to draw attention to the booth, a video describing the DGP in ASL, buccal brushes to illustrate DNA collection, and a binder containing information about genetic counseling and testing using visual aids. DGP used Deaf peer communicators at these events to promote study participation. Direct peer communication can be very effective, especially if the person is seen as credible to the target audience [47], questions can be answered immediately, and the message can be personalized to address particular benefits and barriers important to that person of a similar cultural background. This recruitment channel was used in all four participation locations.

The study team also gave structured informational presentations or staffed an exhibit booth at *organizations* including schools, religious institutions, non-profit organizations e.g., Greater Los Angeles Agency on Deafness and the Hearing Loss Association, and deaf-related conferences e.g., California Association for the Deaf. These venues are a good way to reach professionals in the target group, and DGP’s affiliation with these organizations increased the likelihood that the target audience would pay attention to the message. Moreover, conference sessions can provide information in a memorable way, and materials can be distributed efficiently [38]. This recruitment channel was used in all four study participation locations.

Finally, people are more likely to say yes to a request from someone they know and like [48]. Personal communication can be more effective than other traditional social marketing strategies because of its perceived credibility and impartiality [49]. Through social networks -- “personal media channels” --such as word of mouth from a friend or family member, or through incidental face-to-face contact with a project team member, information about the DGP could happen naturally.

### Data collection

During initial eligibility screening, information about the individual’s primary language and preference for a sign language interpreter when interacting with non-ASL-fluent research staff, available methods for contact, preferred participation location, and primary source for learning about the study (recruitment channel) were recorded. The baseline questionnaire, which was completed immediately following the audiology session, assessed demographic characteristics (age, gender, race/ethnicity, education, income, cultural affiliation) and reasons for genetic testing. Age was dichotomized based on median age of the sample, education was dichotomized as < or ≥4-year college degree, and cultural affiliation was classified as Deaf community, hearing community, both communities, and neither community. Language was categorized as ASL, ASL + English, English, and other (e.g., signed English). As for the group of English-users, they are individuals in this study sample who opted to use the spoken modality with study staff. Methods for contact (price) were coded and dichotomized as sign language-user inclusive (email, videophone, TTY, text only cell phone, pager) or not. Participation location (place) was dichotomized as community-based (CSUN, CSDF, CSDR) or medically-based (UCLA). Participants’ responses to how they learned about the study (promotion) were classified as mass media, community events, organizations, or personal contact. Three potential reasons for genetic testing (product; promotion) are examined (i) ‘to learn why I am deaf/hard-of-hearing’ (a measure of the curiosity messaging); ‘to help research’ (a measure of the generative fulfillment messaging); and ‘to strengthen the Deaf community, ’ (a measure of inclusion of Deaf perspective messaging). For each of these items, respondents rated how strongly they agreed that this was an important reason for genetic testing using a 5-point Likert scale of strongly agree to strongly disagree.

### Analyses

Analyses were performed in this social marketing framework to examine which aspects of communication (price), geography (place), and forms of information exchange (promotion) are important to the culturally and linguistically diverse population of deaf individuals when considering participation in a genetic counseling and testing study. Bivariate associations were assessed using Fisher’s exact test, t-test, or ANOVA. Logistic regression analyses were performed to examine the influence of language on (a) preferred modes of communication between participants and researchers, (b) preference for participation location, and (c) recruitment source. Odds ratios with 95% confidence intervals were also calculated. Linear regression analyses were performed to examine whether the primary and secondary messaging (product; promotion) were associated with participants’ reasons for their interest in genetic testing. Age, gender, education, and recruitment channel were included as covariates as appropriate. Cultural affiliation was not evaluated as a covariate in these analyses because this demographic variable is very strongly associated with language in this sample [30,31]. Participants whose language was described as “other” were excluded from analyses that included language as a variable due to their small sample size and heterogeneous nature. Logistic regression analyses were conducted using the proc LOGISTIC procedure and linear regression analyses were conducted using the proc MIXED procedure in SAS version 9.2 [50]. Statistical significance was defined as p ≤ 0.05.

## Results

### Sample

During a 27 month enrollment period 271 individuals completed an audiology session and 97% were determined eligible for genetic counseling and testing. Among these 271 individuals, 66.1% preferred to use ASL, 19.9% preferred to use ASL + English, 12.6% preferred to use English, and 1.5% used Pidgin Sign Language, Signed English, or another coded language form (other). Sample characteristics of those completing an audiology session are provided in Table 1.

**Table 1:** Sample characteristics (N = 271)

**Characteristic**	**Mean (SD) or No. (%)**
**Language**	
ASL	179 (66.1)
ASL + English	54 (19.9)
English	34 (12.6)
Other	4 (1.5)
**Age, years**	45.8 (15.8)
**Female**	158 (58.3)
**Race/ethnicity**	
Non-Hispanic Caucasian	199 (77.73)
Hispanic	30 (11.72)
Asian	23 (8.98)
Other	4 (1.56)
**≥4-year undergraduate bachelor’s degree**	137 (53.94)
**Median annual income category**	$35,000 - $50,000
**Cultural affiliation**	
Deaf community	140 (55.12)
Deaf and Hearing communities (Bicultural)	90 (35.43)
Hearing community	18 (7.09)
Neither community	6 (2.36)

Participation rate among those eligible to continue onto the genetic counseling and testing phases was very high (Figure 1): 94.3% of those determined eligible attended the pre-test genetic counseling session, 99.6% provided a DNA sample for genetic testing, and 97.6% of those individuals returned to learn their genetic test results. Questionnaire response rates also were high: 98.9% at baseline, 98.8% following pre-test genetic counseling session, 76.8% 1-month following test result disclosure session, and 75.9% 6-months following test result disclosure session. Of note, those who completed the 6-months questionnaire did not differ significantly from who did not complete it in terms of participant language, recruitment channel, or participation location (all p’s > 0.05).

### Price: communication

Linguistic diversity was present in the sample, although most participants preferred to use ASL or ASL + English (Table 1). All participants whose language included ASL, even those who also used spoken English, preferred to interact directly with any non-ASL-fluent research staff via a sign language interpreter, making a sign language interpreter a necessary component of lowering the price of participation. No participant requested a strictly written or lip-reading interaction with research staff. Participants typically used >1 technology for distance communication (Table 2), and technologies varied with preferred language. As shown in Table 3, ASL- and ASL + English-users were more likely to have a videophone (OR = 9.83, 95% CI: 3.26, 29.67; OR = 10.16, 95% CI: 3.06, 33.80, respectively) or a pager (OR = 19.12, 95% CI: 5.42, 67.40; OR = 12.91, 95% CI: 3.42, 48.79, respectively) compared to English-users. In contrast, ASL- and ASL + English-users were less likely to use a voice telephone compared to English-users (OR = 0.12, 95% CI: 0.05, 0.28; OR = 0.09, 95% CI: 0.03, 0.26, respectively). We then classified communication technologies as either sign language inclusive (videophone, pager, TTY, text only cellphone) or not, and found that ASL- and ASL + English-users were more likely to have sign language inclusive technologies for communicating with research staff than English-users (OR = 35.18, 95% CI: 11.96, 103.42; OR = 21.96, 95% CI: 6.37, 75.65, respectively).

**Table 2:** Distribution of communication technologies, participation locations, and recruitment channels by participant language

	**Language group**			
**Characteristic**	**ASL**	**ASL + English**	**English**	**Entire sample**^**a**^
**N**	179	54	34	271
**Communication technologies (%)**				
Email	93.3	100	100	95.6
Videophone	62.0	62.9	11.8	55.7
Pager	60.3	59.3	11.8	53.5
Telephone	21.8	16.7	70.6	26.6
TTY	5.6	7.4	8.8	7.0
Text cell phone	2.2	9.3	5.9	4.1
Voice to text phone	0	0	2.9	0.4
Sign language inclusive	90.5	88.9	26.5	82.0
**Participation locations (%)**				
University of California Los Angeles	38.6	33.3	64.7	40.6
California State University Northridge	20.1	33.3	20.6	23.6
California School for the Deaf Fremont	25.1	18.5	11.8	21.8
California School for the Deaf Riverside	16.2	14.8	2.9	14.0
**Recruitment channels (%)**				
Community events	46.9	42.9	3.6	41.2
Organizations	27.4	24.3	42.9	28.4
Personal media	21.3	32.1	26.5	23.9
Mass media	4.3	6.1	21.4	6.6

^a^Includes n = 4 who used other communication forms, e.g., signed English, Pidgin Sign English.

**Table 3:** Logistic regression analyses: effect of participant language on communication technologies, participation location, and recruitment channel

**Social marketing mix**		**Language**^**a**^		**Education**^**a**^	**Age**^**a**^	**Gender**^**a**^
**Concept**	**Outcome variable**	**ASL Odds ratio (95% CI**^**b**^**)**	**ASL + English Odds ratio (95% CI)**	**≥ BA degree Odds ratio (95% CI)**	**>44 yrs Odds ratio (95% CI)**	**Female Odds ratio (95% CI)**
**Price: communication technology**	Video phone	9.83	10.16	1.50	0.73	1.35
		(3.26, 29.67)	(3.06, 33.80)	(0.88, 2.56)	(0.43, 1.26)	(0.79, 2.33)
	Pager	19.12	12.91	2.28	1.12	0.84
		(5.42, 67.40)	(3.42, 48.79)	(1.31, 3.97)	(0.65, 1.93)	(0.49, 1.46)
	Voice telephone	0.12	0.09	1.17	0.93	1.12
		(0.05, 0.28)	(0.03, 0.26)	(0.64, 2.14)	(0.51, 1.72)	(0.61, 2.06)
	Sign language inclusive	35.18	21.96	1.18	3.88	0.70
		(11.96, 103.42)	(6.37, 75.65)	(0.51, 2.74)	(1.60, 9.43)	(0.31, 1.59)
**Place: participation location**	Community-based^c^	2.72	3.39	0.96	1.42	1.08
		(1.20, 6.14)	(1.31, 8.74)	(0.57, 1.62)	(0.84, 2.37)	(0.66, 1.62)
	Community-based^d^	1.49	3.01	0.83	1.43	0.90
		(0.53, 4.17)	(0.95, 9.51)	(0.41, 1.68)	(0.73, 2.81)	(0.45, 1.79)
**Promotion: recruitment channel**	Community event^e^	23.18	19.63	--	--	--
		(3.60, 976.77)	(2.69, 879.57)	--	--	--
	Organization	0.69	0.52	1.31	0.65	1.17
		(0.27, 1.74)	(0.17, 1.61)	(0.67, 2.54)	(0.34, 1.24)	(0.62, 2.23)
	Personal media	0.51	0.64	1.75	0.84	0.93
		(0.20, 1.32)	(0.21, 1.92)	(0.86, 3.54)	(0.43, 1.64)	(0.47, 1.84)
	Mass media	0.13	0.22	0.59	1.91	1.02
		(0.04, 0.48)	(0.05, 1.04)	(0.20, 1.77)	(0.63, 5.80)	(0.34, 3.09)

^a^Reference group for: Language = English-users; Education = < BA degree; Age = < 44 years; Gender = Male.

^b^CI = Confidence Interval.

^c^Odds ratios = odds of participating at community-based location rather than medically-based location.

^d^Odds ratios = odds of participating at California State University Northridge rather than University of California Los Angeles.

^e^Unadjusted exact logistic regression analysis was performed due to small numbers of English-users.

### Place: participation locations

The participation sites varied in total number of months of operation with UCLA and CSUN fully operational for 27 months, CSDF for 15 months, and CSDR for 8 months. On average, 2.3 – 4.2 individuals were enrolled per month of operation at each location. Monthly rate of enrollment did not differ significantly across the four locations (F(3,77) = 1.92, p = 0.13). Table 2 gives the distribution of participation locations by preferred language.

We investigated the effect of language on choice of participation location. As shown in Table 3, ASL- and ASL + English-users were more likely to enroll in a community-based location (OR = 2.72, 95% CI: 1.20, 6.14; OR = 3.39, 95% CI: 1.31, 8.74, respectively) compared to English-users who preferred the medically-based location. Specifically, 62.7% (146/233) of participants who preferred to use ASL or ASL + English enrolled at the community-based locations; in contrast, 64.7% (22/34) of English-users enrolled at the medically-based location. A similar trend was noted when we restricted our analysis to enrollment at the community-based location (CSUN) and medically-based location (UCLA) sited in the same geographic area, although it failed to achieve statistical significance (ASL-users OR = 1.49, 95% CI: 0.53, 4.17; ASL + English-users OR = 3.01, 95% CI: 0.95, 9.51).

### Promotion: recruitment channels

We implemented 49 distinct recruitment activities during the 25 month recruitment period. Over half (55.1%) of recruitment activities focused on staffing an exhibit booth or giving presentations at events/meetings/conferences sponsored by organizations, 24.5% on staffing an exhibit booth or giving presentations at community events such as DeafNation Expos, and 20.4% on the use of mass media channels. Although only a quarter of recruitment activities focused on community events, this was the most frequently reported channel (41.2%) for learning about the study. From a social marketing perspective, this result indicates that researchers should increase emphasis on community events in any future genetic studies in the Deaf community. Table 2 gives the distribution of recruitment channels by preferred language.

We investigated the effect of language on recruitment channel (mass media, community events, organizations, personal media). For these analyses we focus on those who participated at UCLA, CSUN, and CSDF because all four recruitment channels were used in the geographic areas of these participation locations. As shown in Table 3, ASL-users and ASL + English-users were more likely to be recruited through community events than English-users (OR = 23.18, 95% CI: 3.60, 976.77; OR = 19.63, 95% CI: 2.69, 879.57, respectively). In fact, 47.45% (65/137) of ASL-users and 43.9% (18/41) of ASL + English-users were recruited through community events compared to 3.7% (1/27) of English-users. In contrast, ASL- and ASL + English-users were less likely than English-users to be recruited through mass media (OR = 0.13, 95% CI: 0.04, 0.48; OR = 0.22, 95% CI: 0.05, 1.04, respectively), with 5.11% (7/137) of ASL-users and 7.32% (3/41) of ASL + English-users compared to 22.22% (6/27) of English-users. Language was not significantly associated with recruitment through organizational channels or personal media (Table 3) and roughly half of the participants were recruited through these two channels (Table 2).

### Promotion: messaging

We found that 91.7% strongly agreed/agreed that they were interested to learn why they are deaf, and 91.6% strongly agreed/agreed that they were interested in helping research, suggesting that their reasons for genetic testing reflected the curiosity and generative fulfillment primary recruitment messaging. Linear regression analyses demonstrated that the primary messaging resonated with all language groups and through all recruitment channels since neither of these variables were significant predictors of participants’ responses to these genetic testing reasons (p’s > 0.05). We explored whether participants’ genetic testing reasons reflected the secondary messaging that highlighted inclusion of the Deaf perspective in the research process and found that 75.2% of participants strongly agreed/agreed that they were interested in genetic testing to strengthen the Deaf community. Response to this item was significantly associated with language (p = 0.006) and recruitment channel (p = 0.005). Specifically, ASL- and ASL + English-users indicated stronger agreement compared to English-users; and those who learned of the study through community events, organizations, and personal media indicated stronger agreement compared to those who learned of the study through mass media.

## Discussion

Recruiting deaf individuals to participate in health services research has been challenging. Studies suggest that a variety of social, cultural, and linguistic factors that have resulted in the exclusion of deaf individuals from the bulk of health services research must be addressed to overcome recruitment barriers [7]. This article demonstrates the value of explicitly addressing social exclusion and social cohesion in a health service research design to attract the deaf population’s participation, and ultimately to reduce health disparities. Moreover, we demonstrate that these factors can be addressed within a social marketing framework, and that this framework helps assess whether recruitment of an underserved, hard-to-reach population is successful.

Recruitment in the DGP was quite successful. Not only did we achieve our target sample size of 250, but 97% of individuals who attended an audiology session were eligible for the genetic counseling and testing sessions. This result demonstrates that our recruitment and initial eligibility screens effectively targeted the desired study population. Moreover, participant retention was very high, where >90% of eligible participants submitted a DNA sample for genetic testing during pre-test genetic counseling and returned to learn their genetic test results, and 76% of participants responded to the final 6 months post results questionnaire. Importantly, participants retained out to 6-months post-test result disclosure did not differ significantly from those not retained across recruitment channel, participant language, or participation location. These results not only demonstrate that our study design was flexible enough that those recruited felt committed to their participation, but that this study design did not produce a “missingness” bias into the questionnaire data. Given reports that few deaf individuals, particularly sign language-users are seen in genetics clinics [51], these results are highly encouraging. By evaluating recruitment in a social marketing framework, we can specifically determine what aspects of our study design are effective for conducting genetics health service research in the deaf population and the Deaf community and determine how to generalize these aspects to health service research in underserved minority groups.

We utilized a social marketing framework to identify the components of the social marketing mix used in our study design. From this analysis we interpret our study design as reducing the participation “price” by reducing language barriers and attending to the cultural and linguistic differences between researchers and participants that contribute to social exclusion and lack of social cohesion, i.e., by employing a variety of communication technologies, certified sign language interpreters, and culturally sensitive genetic counselors, and by providing survey questionnaires in ASL. Consistent with other studies, e.g., [8,9,11], our results demonstrate that ensuring communication between deaf individuals and researchers is an effective means for engaging them in a research project. Furthermore, our findings demonstrate the importance of attending to the linguistic diversity of the deaf population as well as language concordance/discordance between researchers and participants. Videophone and sign language interpreters were used for communication between ASL- and ASL + English-users and non-ASL fluent study personnel while voice telephone and no interpreters were preferred by deaf English-users for interactions with English-using project personnel. Ironically, we faced initial institutional reluctance in the medically-based location to allow videophone use, an internet-based communications device analogous to videoconferencing, due to local interpretation of the U.S. Health Insurance Portability and Accountability Act (HIPAA) regulations which protects the privacy of individually identifiable health information. Videophones are an effective way to remove communication barriers [7] but they require open and non-firewalled connection, which raised the potential for an IP security issue. Although a recent review did not reveal a negative effect of HIPAA on minority health research [1], our experience reveals a lack of cultural awareness in medical institutions of the importance of videophone as a communication device for ASL-users, which could negatively affect health service research in this population. Concerns about violating HIPAA should be eliminated if the deaf person gives their approval to go ahead after appropriate consent.

We can also interpret our results as addressing social inclusion and social cohesion through the “place” element of social marketing. We engaged in statewide recruitment where there are deaf residential schools and deaf organizations, and thus provided three community-based participation locations along with a medically-based location. Previous health-related studies have used community partnerships [10,11], and we found that developing these partnerships was highly effective for engaging sign language-users with this research. Moreover, because we analyzed participation location by preferred language, our results provide even stronger evidence for the importance of “place”. Not only were ASL- and ASL + English-users more likely to be enrolled at the community-based locations than English-users, but nearly two-thirds of ASL- and ASL + English-users in our sample opted to participate through a community-based location. Hence, using community-familiar locations where sign language-users are concentrated enhanced desirability to participate in health service research. Furthermore, when controlling for geographic location, a similar non-significant trend was observed. Lack of statistical significance may reflect lack of power with the reduced sample size or that efforts to create a culturally and linguistically appropriate environment for ASL-users at the medically-based location were effective to engage sign language-users. Together, these results suggest that culturally and linguistically appropriate environments - which typically are part of the fabric of community-based locations - can be achieved in other locations and effectively engage sign language-users in health service research. Thus, endeavors at developing culturally and linguistically appropriate genetics health services and health service research for the ASL-using community should use a two-pronged approach by focusing efforts on locations with a heavy concentration of ASL-users, such as nearby deaf residential schools or Deaf community service organizations, and creating culturally and linguistically appropriate environments in medically-based settings.

In terms of “promotion,” participants were recruited from all four channels. However, deaf English-users were more likely to learn about the study through mass media, while community events were the most effective strategy for recruiting sign language-users. This result also demonstrates the importance of attending to the diversity of the deaf population. Community events are a common site of recruitment for studies involving Deaf individuals [8,11,12,52,53] and our study suggests that its effectiveness exceeds other recruitment channels. The importance of community events for recruiting sign language-users is intriguing because a recent review of recruitment strategies for enrolling cultural and linguistic minorities into health service research found that although outreach through community events was a frequent strategy, it was the least successful approach in 77% of 42 studies that employed it [3]. We hypothesize several reasons for our success with community events. First, the Deaf community’s collective nature and desire to socialize with other ASL-users [54] facilitate both the construction of, and attendance at, Deaf-related community events. Secondly, community events offered opportunities for flexible, interpersonal connections between project personnel and potential participants, involved peer communicators (ASL-users) which increased credibility and trust, and demonstrated our commitment to inclusivity by showing collaboration between Deaf and hearing researchers. Furthermore, attending community events demonstrated that research personnel were willing to meet Deaf people in their preferred environments in order to develop trust and rapport. Anecdotally, individuals approaching our exhibit booth at community events frequently had questions regarding DNA sample ownership and this recruitment venue allowed us to explain that the sample would be discarded at the study’s conclusion. It is possible that this practice also served to increase trust and build rapport and thereby increased a sense of social inclusion because community members believed that the program focused on their unique needs [7,27]. Our experience also demonstrates that deaf English-users are a challenging group to reach directly and more research is needed to identify successful recruitment strategies for this group.

We interpret DGP’s core product as increasing awareness about genetic counseling and testing and the actual product to determine if genetics explains why a person is deaf (curiosity) and to provide an opportunity to educate society-at-large about what genetic testing means to deaf individuals and the Deaf community (generative fulfillment), i.e., giving this group a chance to express their views to the general population through this research. Those were primary recruitment messages and the majority of participants, regardless of preferred language or recruitment channel, endorsed those ideas as their participation reasons. Thus, curiosity and generative fulfillment messaging were successful messaging techniques. Generative fulfillment has been successful in other social marketing campaigns [23], and these findings extend this technique’s effectiveness to recruiting deaf individuals into health service research. Results also suggest that our secondary messaging indicating inclusion of the Deaf perspective would be an effective technique for specifically engaging ASL-users and that this messaging technique would be most effective via community events, organizations, and personal media.

This study has several limitations. Only one recruitment channel was recorded for most participants, although participants might have learned of the DGP through multiple venues. Thus, although it appears that our mass media campaign was least effective for recruiting participants, this channel might have increased effectiveness of the other recruitment channels. We also could not record data on individuals who screened ineligible, thus we cannot comment on each recruitment channel’s relative effectiveness to target eligible participants. Furthermore, given the use of the internet, email listservs, conference presentations, and exhibit booths, it was not possible to track how many individuals were reached through our recruitment methods and channels. Thus, another limitation of this study is that we are unable to comment on the response rate produced by our recruitment approach. In addition, the individuals who chose to participate in the DGP may differ in important ways from those who did not participate. As one example, our study participants were more highly educated and had a higher median income than a national sample of prelingually deaf adults [55]. As another example, deaf individuals attending community events (our major recruitment source) may not be typical of the larger deaf population. Hence, generalizing our findings to the general deaf population should be done with caution.

## Conclusions

This paper interprets a study design through a social marketing framework and provides a guide of how to conceptualize, implement and assess a social marketing approach to genetics health services research for the deaf population. Using the social marketing framework allowed us to determine that our study effectively engaged deaf individuals, particularly sign language-users, and suggests that all genetics health service research designs should address social inclusion and cohesion. Results also suggest that the deaf population’s cultural and linguistic diversity, geography, and forms of information exchange must be taken into account in study designs for successful recruitment. A social marketing approach that incorporates critical social determinants of health provides a novel and important framework for genetics health service research targeting specific, and hard-to-reach, underserved groups.

## Endnotes

^a^The general consensus of the wording usage for various members of our study population is somewhat in flux. Hence for this study, the usage of ‘deaf’ lexicon with lowercase ‘d’ is applied throughout this article to reflect this study population in a broader sense. The term deaf applies to individuals ranging from culturally Deaf, deaf, hard-or-hearing either using sign language or using residual hearing by speech [21]. The usage of ‘Deaf’ with uppercase ‘D’ as widely adopted from Woodward’s convention [56] is used here to exemplify a targeted group of our study who use sign language and identify themselves as a part of Deaf community.

## Abbreviations

ASL: American Sign Language; ANOVA: Analysis of variance; CI: Confidence interval; CSDF: California School for the Deaf – Fremont; CSDR: California School for the Deaf – Riverside; CSUN: California State University Northridge; DGP: Deaf Genetics Project; DNA: Deoxyribonucleic acid; HIPAA: Health Insurance Portability and Accountability Act; OR: Odds ratio; TTY: Text telephone; UCLA: University of California Los Angeles.

## Competing interests

The authors have no competing interests to declare.

## Authors’ contributions

YK conceptualized this manuscript, contributed to the analysis and interpretation of data, and wrote the first draft. PB contributed to the analysis and interpretation of data and to revisions of the manuscript. KH carried out the statistical analysis. JS contributed to the analysis and interpretation of data and to revisions of the manuscript. CP made contributions to the conception and design of this study, to the analysis and interpretation of data and to revisions of the manuscript. All authors read and approved of the final manuscript.

## Pre-publication history

The pre-publication history for this paper can be accessed here:

http://www.biomedcentral.com/1471-2288/13/145/prepub
